# Trends and predictors of early ablation for Atrial Fibrillation in a Nationwide population under age 65: a retrospective observational study

**DOI:** 10.1186/s12872-020-01446-9

**Published:** 2020-04-06

**Authors:** Robert N. D’Angelo, Rahul Khanna, Robert W. Yeh, Laura Goldstein, Iftekhar Kalsekar, Stephen Marcello, Patricia Tung, Peter J. Zimetbaum

**Affiliations:** 1 Richard A. and Susan F. Smith Center for Outcomes Research, Division of Cardiology, Beth Israel Deaconess Medical Center, 185 Pilgrim Road, Boston, MA 02215 USA; 2 Medical Device Epidemiology and Real World Data Science, Johnson and Johnson, New Brunswick, NJ USA; 3 Franchise Health Economics and Market Access, Johnson and Johnson, New Brunswick, NJ USA; 4 Medical Safety, Johnson and Johnson, New Brunswick, NJ USA

**Keywords:** Atrial fibrillation, Catheter ablation, Rhythm control

## Abstract

**Background:**

Catheter ablation (CA) has emerged as an effective treatment for symptomatic atrial fibrillation (AF). However practice patterns and patient factors associated with referral for CA within the first 12 months after diagnosis are poorly characterized. This study examined overall procedural trends and factors predictive of catheter ablation for newly-diagnosed atrial fibrillation in a young, commercially-insured population.

**Methods:**

A large nationally-representative sample of patients age 20 to 64 from years 2010 to 2016 was studied using the IBM MarketScan® Commercial Database. Patients were included with a new diagnosis of AF in the inpatient or outpatient setting with continuous enrollment for at least 1 year pre and post index visit. Patients were excluded if they had prior history of AF or had filled an anti-arrhythmic drug (AAD) in the pre-index period.

**Results:**

Early CA increased from 5.0% in 2010 to 10.5% in 2016. Patients were less likely to undergo CA if they were located in the Northeast (OR: 0.80, CI: 0.73–0.88) or North Central (OR: 0.91, CI: 0.83–0.99) regions (compared with the West), had higher CHA_2_DS_2_-VASc scores, or had Charlson Comorbidity Index (CCI) score of 3 or greater (OR: 0.61; CI: 0.51–0.72).

**Conclusions:**

CA within 12 months for new-diagnosed AF increased significantly from 2010 to 2016, with most patients still trialed on an AAD prior to CA. Patients are less likely to be referred for early CA if they are located in the Northeast and North Central regions, have more comorbidities, or higher CHA_2_DS_2_-VASc scores.

## Background

Atrial fibrillation (AF) can present with severe symptoms, thromboembolic events, and hemodynamic instability that leads to morbidity and mortality and frequent hospitalizations [1–4]. Patients who are highly symptomatic from AF are candidates for a rhythm control strategy. Rhythm control strategies have traditionally focused on anti-arrhythmic drugs (AADs), although studies have shown that these drugs have numerous side effects and many patients do not durably maintain sinus rhythm [1, 5, 6]. Catheter ablation (CA) has emerged as a viable alternative to AADs that may better maintain sinus rhythm or reduce AF burden.

Emerging evidence has suggested that CA may be more effective than AADs for improving symptoms of AF, although the effect of CA on hard patient outcomes including hospitalization, adverse events, and mortality is unclear. Several randomized controlled trials have sought to assess the effectiveness of CA as a first-line therapy for AF and found CA resulted in decreased AF burden and improved subjective quality of life compared to AADs [7–10]. A recent retrospective study of younger, commercially-insured patients found that CA resulted in fewer hospitalizations for AF and heart failure [11]. Additionally, there are several ongoing clinical trials to assess the safety and efficacy of early ablation for AF in patients who have not been treated with AADs (NCT03118518, NCT02686749).

These studies have not addressed the optimal timing of CA, although a prospective study showed that shorter duration between AF diagnosis and CA reduced rate of AF recurrence and adverse cardiac remodeling, using NT-proBNP and left atrial size as surrogate measures [12]. In particular, patients who underwent CA within 1 year of AF diagnosis (referred to as “early CA” for study purposes), had the greatest chance of long-term maintenance of sinus rhythm. Yet, recent consensus guidelines continue to recommend trial of Class I or Class III anti-arrhythmic prior to CA for AF [13]. Additionally, many US payer-based guidelines mandate treatment with an antiarrhythmic drug prior to referral for catheter ablation, which may delay time to CA and reduce its long-term effectiveness.

It is not well known how this information has translated to real-world practice, particularly with regard to how the frequency of early CA is changing over time, regional differences in practice, and patient specific factors that lead to referral for early CA. This study assesses practice patterns for treatment of atrial fibrillation within a young nationally-representative and commercially-insured population.

## Methods

### Study population

A retrospective observational study was conducted using medical and prescription claims data from the IBM MarketScan® Commercial Database. The Commercial database includes a nationally-representative, Health Information Portability and Accountability Act of 1996 (HIPAA) compliant sample of patients with employer-sponsored private health insurance [14]. The study was exempt from Institutional Review Board approval at Beth Israel Deaconess Medical Center.

Patients were identified for inclusion in the study using *International Classification of Diseases, 9th revision and 10th revision*, *Clinical Modification* (ICD-9/ICD-10) diagnostic codes for AF (427.21, I48.X). Patients age 20 to 64 with at least two different visits either in the inpatient or outpatient setting with a primary diagnosis of AF within 3 months from January 1, 2010 to September 30, 2016 were included. The use of 2 different visits was used in order to increase the specificity of the AF diagnosis. The date of first AF diagnosis was considered as ‘index AF diagnosis’. Patients needed to be continuously enrolled for at least 12 months pre-index and 12 months post-index period to be included. Because the goal was to diagnose only those patients with new onset atrial fibrillation, patients were excluded if they had any diagnosis of AF in the pre-index period or if they had filled an AAD in the pre-index period. The following AADs were identified: amiodarone, disopyramide, dofetilide, dronedarone, flecainide, quinidine, propafenone, and sotalol.

### Covariates and outcomes

Patient demographics included age, sex, region (Northeast, North Central, South, and West), insurance type (comprehensive, Exclusive Provider Organization (EPO) or Health Maintenance Organization (HMO), Point of Service (POS) with capitation, Preferred Provider Organization (PPO), Consumer-Drive Health Plan (CDHP) or High-Deductible Health Plan (HDHP)). Patient clinical characteristics included Charlson Comorbidity Index (CCI), CHA_2_DS_2_-VASc Score, and previously defined Elixhauser comorbidities [15–17]. The primary outcome of interest was CA within the first year after AF diagnosis, identified using the following ICD-9/ICD-10 codes (ICD-93734; ICD-1002553ZZ, 02563ZZ, 02573ZZ, 02583ZZ, 025K3ZZ, 025L3ZZ, 025M3ZZ, 025S3ZZ, 025T3ZZ) and CPT codes (93,651, 93,656). Additional outcome variables included number of AADs trialed, anticoagulants used, and Direct Current Cardioversions (DCCV). The following oral anticoagulants were included: warfarin, apixaban, dabigatran, rivaroxaban, and edoxaban.

### Statistical analysis

We first examined rates of CA within the first year of index for each year of the study, as well as preceding AAD use, and examined temporal trends using an XYZ test. We then developed a multivariable logistic regression model to determine factors associated with CA within the first year. Covariates included patient demographics, CCI Score, CHA_2_DS_2_-VASc score, and comorbidities. Results are presented as odds ratios with 95% confidence intervals. All analyses were performed with SAS for Windows, version 9.4 at a 2-tailed significance of *P* < 0.05.

## Results

### Clinical characteristics

**Fig. 1:**
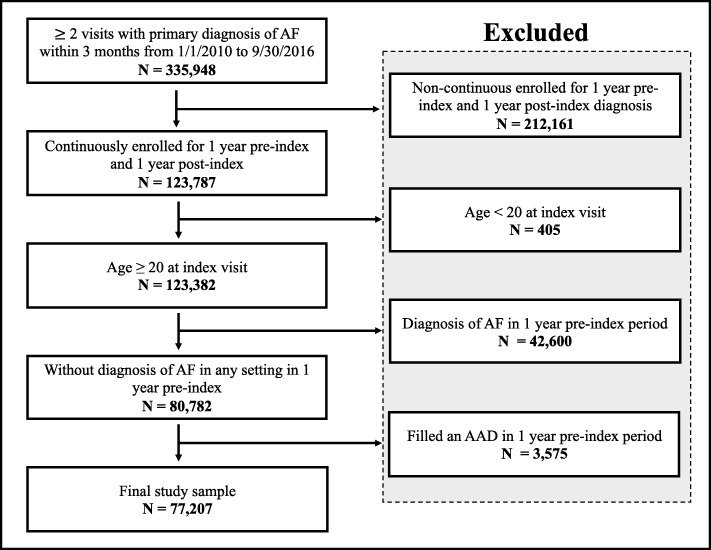
Central Illustration: Flow diagram - Selection of study participants from years 2010–2016

Initial and final sample sizes after applying study inclusion and exclusion criteria are shown in Fig. 1. Of 335,948 patients who met initial screening criteria, 77,207 patients with a new diagnosis of AF were included in the final study sample. Characteristics of patients included in the study are presented in Table 1. Mean age was 58.8 (± 8.4), 67% were male, and the majority (77%) were diagnosed in the outpatient setting. The cohort drew most heavily from the South (38%), followed by North Central (25%), Northeast (19%), and West (16%), reflective of data-sharing agreements with commercial insurance plans rather than prevalence of atrial fibrillation within these regions. Most patients were insured by PPO health plans (61%), followed by EPO/HMO (13%), with other insurance types less frequently represented.

**Table 1:** Baseline patient characteristics

Characteristic	Patients (*n* = 77, 207)
Age, mean (SD)	53.8 (8.4)
Male (%)	51,570 (66.8)
US geographic region, n (%)	
Northeast	14,324 (18.6)
North Central	19,436 (25.2)
South	29,661 (38.4)
West	12,634 (16.4)
Unknown	1152 (1.5)
Index Diagnosis, n (%)	
Inpatient	17,434 (22.6)
Outpatient	59,773 (77.4)
Insurance Type, n (%)	
Comprehensive or EPO or HMO	13,627 (17.7)
POS & POS with capitation or PPO	54,306 (70.3)
CDHP or HDHP	8145 (10.6)
Unknown	1129 (1.5)
CCI^a^, n (%)	
Score 0	45,921 (59.5)
Score 1–2	23,126 (30.0)
Score ≥ 3	8160 (10.6)
CHA_2_DS_2_-VASc, n (%)	
Score 0	23,675 (30.7)
Score 1–2	42,588 (55.2)
Score ≥ 3	10,944 (14.2)
Comorbidity, n (%)	
Sleep apnea	8625 (11.2)
Obesity	7558 (9.8)
Diabetes	13,651 (17.7)
Hypertension	35,658 (46.2)
Congestive heart failure	4964 (6.4)
Cardiomyopathy	2637 (3.4)
Chronic pulmonary disease	9333 (12.1)
Renal disease/failure	2519 (3.3)
Other arrhythmia	9766 (12.7)
Wolf-Parkinson-White Syndrome	81 (0.1)
Non-paroxysmal AV nodal tachycardia	86 (0.1)
Paroxysmal supraventricular tachycardia	1903 (2.5)
Atrial flutter	2213 (2.9)
Valvular heart disease	7178 (9.3)
Congenital heart disease	718 (0.9)
Hyperthyroidism	613 (0.8)
Ischemic heart disease	4584 (5.9)

^a^*CCI* Charleston Cormorbidity Index

Almost 60% of patients had a CCI score of 0. Fifty-five percent of patients had a CHA_2_DS_2_-VASc score of 1–2, 31% had a score of 0, and the remaining 14% had a score of 3 or higher. The most common comorbidity was hypertension (46%), followed by diabetes (18%). Other clinical characteristics are presented in Table 1.

Twenty-nine percent of patients were trialed on an AAD, and 46% were started on an anticoagulant within first year of incident AF diagnosis. While 24% of patients underwent DCCV, only 7% of patients underwent CA. Additional outcomes are presented in Table 2. For those undergoing CA in the first year, 59% of patients were trialed on an AAD prior to undergoing CA, which increased over the study period from 58% in 2010 to 64% in 2016.

**Table 2:** Primary outcomes – Antiarrhythmic medications, anticoagulant medications, cardioversions, and catheter ablation

Outcomes	Patients (n = 77,207)
Antiarrhythmic medication^a^, n (%)	
0	55,077 (71.3)
1	19,196 (24.9)
2+	3,934 (3.8)
Anticoagulant medication^b^, n (%)	
0	41,479 (53.7)
1	32,239 (41.8)
2+	3,489 (4.5)
Direct current cardioversion, n (%)	18,875 (24.4)
Catheter ablation, n (%)	5,451 (7.1)

^a^ Anti-arrhythmic medications included: amiodarone, disopyramide, dofetilide, dronedarone, flecainide, quinidine, propafenone, and sotalol

^b^ Anticoagulant medications included: warfarin, apixaban, dabigatran, rivaroxaban, and edoxaban

**Fig. 2:**
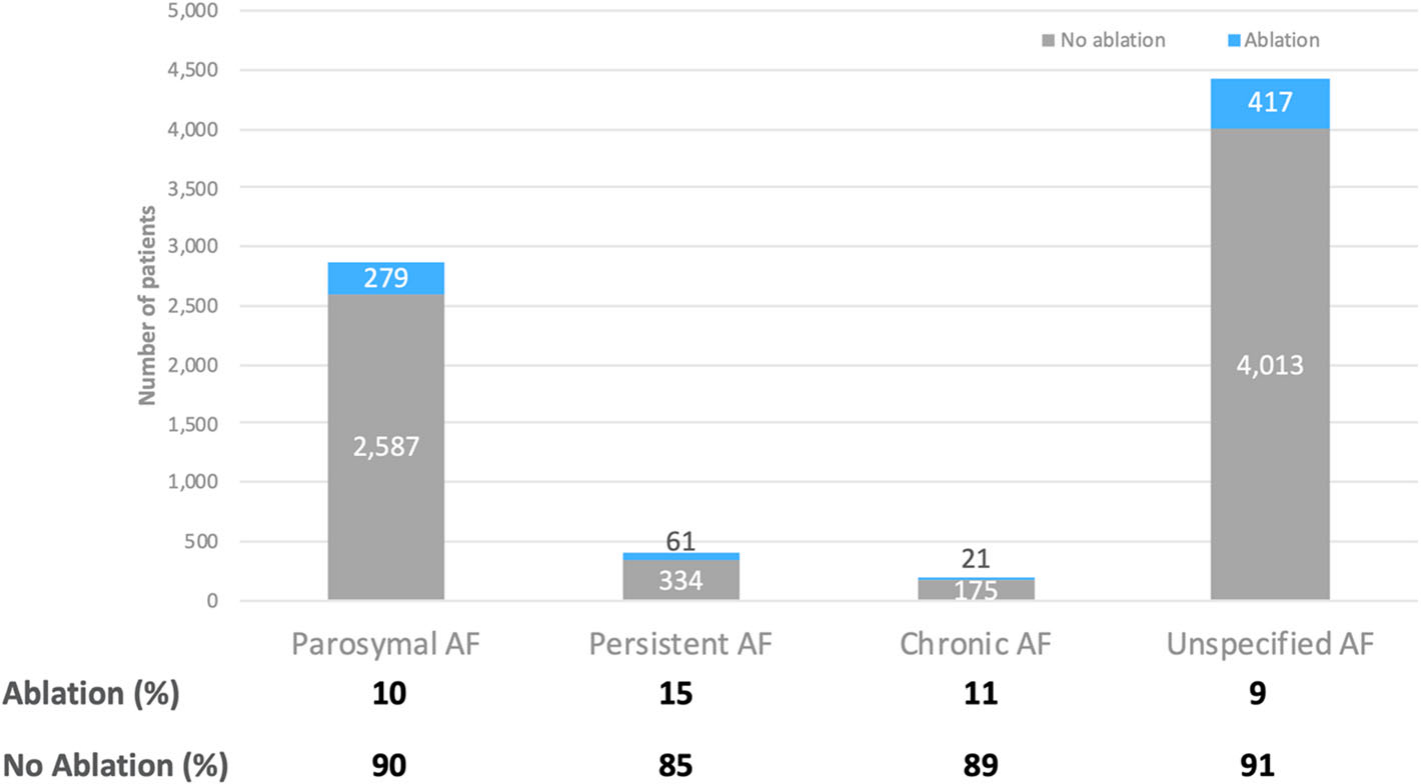
Classification and Rates of Ablation by ICD-10 AF Subtype

After implementation of ICD-10 in 2015, the following AF subtypes were recorded: paroxysmal, persistent, chronic, and unspecified. The majority of patients were classified as having either paroxysmal (36%) or unspecified (56%) AF, with persistent (5%) and chronic (2%) AF rarely represented. The overall prevalence of ablation within these groups was similar, ranging from 9% in unspecified AF to 15% for persistent AF, as shown in Fig. 2.

### Trends and predictors of early CA

**Fig. 3:**
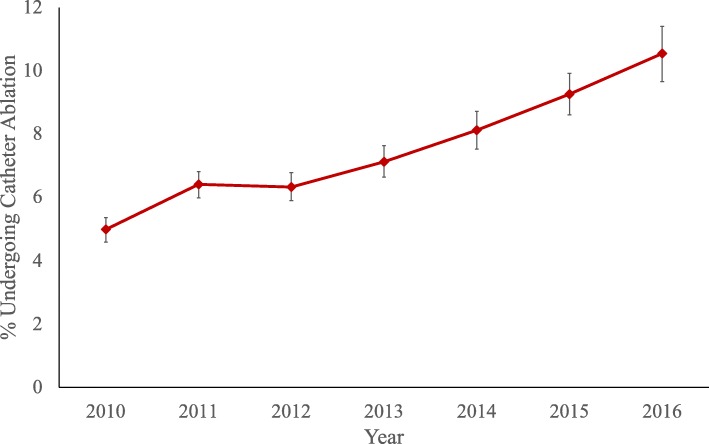
Growth in early catheter ablation for AF from 2010 to 2016

The proportion of patients who underwent early ablation increased steadily from 5.0% in 2010 to 10.5% in 2016. Temporal trends in early CA are presented in Fig. 3. The odds of undergoing CA within the first year increased significantly over the study period, with patients in 2016 2.2 times more likely to undergo CA than those in 2010 (Odds ratio [OR]: 2.18; 95% Confidence Interval [CI]: 1.93–2.46). Patients were less likely to undergo CA if they were located in the Northeast (OR: 0.80, CI: 0.73–0.88) or North Central (OR: 0.91, CI: 0.83–0.99) regions (reference ‘West’). Insurance type also affected likelihood of undergoing CA, as patients with PPO (OR: 1.09; CI: 1.01–1.18) or high deductible health plans (OR: 1.16; CI: 1.04–1.29) were more likely to undergo CA.

**Fig. 4:**
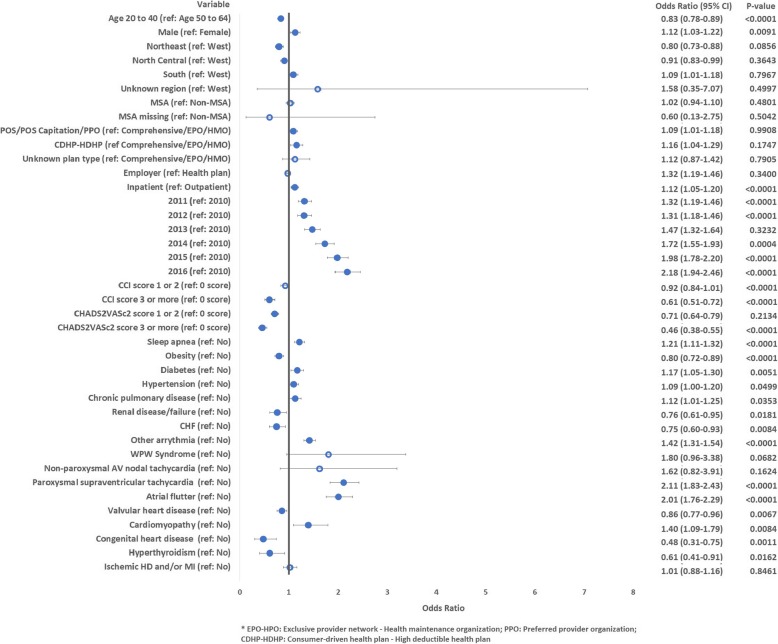
Predictors of catheter ablation within 1 year after diagnosis of atrial fibrillation

A number of patient-specific factors were predictive of undergoing early CA. Overall, patients with more comorbidities were less likely to undergo CA. Specifically, patients with CCI score of 3 or greater (OR: 0.61, CI: 0.51–0.72; reference score 0) or CHA_2_DS_2_-VASc score greater than 0 were significantly less likely to undergo CA. Patients with CHA_2_DS_2_-VASc scores of 3 or greater were least likely to undergo ablation (OR: 0.46; CI: 0.38–0.55; reference score 0). Patients were more likely to undergo ablation if they were male (OR: 1.12, CI: 1.03–1.22) or had cardiomyopathy (OR: 1.40, CI: 1.09–1.79). Patients with atrial flutter were two times more likely to undergo CA (OR: 2.00, CI: 1.75–2.29), while those with other arrhythmias were 41% more likely to undergo CA in the first-year post-incident diagnosis of AF (OR: 1.41, CI: 1.30–1.53). Additional factors predictive of early ablation are presented in Fig. 4.

## Discussion

A number of studies have demonstrated the benefits of early referral for CA in selected patient populations. Single-center studies and randomized-controlled trials have shown that early ablation can lead to improvements in adverse cardiac remodeling, greater success in maintaining sinus rhythm, and decreased need for repeat CA [12, 18]. Studies have shown that young patients may benefit the most from early ablation, however most observational and retrospective studies have focused on patients older than 65 [19–21]. Our study addresses trends in catheter ablation within a young population and specifically identifies predictors of referral for early ablation.

Despite recognition of the benefits of an early rhythm control strategy in some patients, only 30% of patients were trialed on an anti-arrhythmic, and an even smaller portion, 7%, were referred for early CA. This may reflect low arrhythmia burden of newly-diagnosed AF within a young population, or a missed opportunity to avoid long-term sequelae of undertreated AF. Nonetheless, the odds of being referred for early ablation increased by 2.2 times over the study period. This suggests the increases in CA shown in other studies are not simply being driven by increased referral of older patients or those who have already been trialed on AADs. Although we were unable to directly measure AF burden, patients diagnosed in the inpatient setting were 12% more likely to be referred for CA, suggesting severity of AF symptoms were a predictor of early CA.

As AF subtype was recorded after implementation of ICD-10, we investigated whether this affected ablation strategy. We would expect that patients with paroxysmal AF or persistent AF of short duration would be most likely to benefit from ablation and therefore would be referred at higher rates [12, 22–24]. Yet, the rates of ablation were similar, likely due to inclusion and exclusion criteria that minimized the number of patients with long-standing persistent AF or chronic AF and coding integrity. Overall, the ability to generalize outcomes by AF subtype is limited given a large proportion of patients in the post ICD-10 era are classified as having unspecified AF.

Forty-two percent of patients proceeded to ablation without trialing an AAD in 2010, which decreased to 36% in 2016. The increase in AAD use prior to ablation likely reflects increased recognition of the benefits of rhythm control in a young population along with insurance mandates to trial an AAD prior to ablation. As there is increasing recognition that many patients do not have durable responses to treatment with AADs, proceeding with early ablation can lead to improved outcomes and avoid likelihood of repeat ablation [25]. Overall, these findings are compatible given that many younger patients have a short duration of treatment with AADs prior to ablation [20].

Overall, healthier patients were most likely to be referred for ablation, both reflected in CHA_2_DS_2_-VASc score and CCI. Patients with lower CHA_2_DS_2_-VASc scores were more likely to undergo ablation, which could reflect decreased perceived procedural risk or the desire to discontinue anticoagulation after ablation. Further work is required to understand whether patients are discontinuing anticoagulation after ablation. Similar to results found in other studies, fewer patients are treated with anticoagulants than is recommended by guidelines. Only 46% of patients with a CHA_2_DS_2_-VASc score of 1–2 and 60% of patients with a CHA_2_DS_2_-VASc score of 3 or higher filled a prescription for an anticoagulant in the year after diagnosis. While most comorbidities were negatively associated with early CA, patients with cardiomyopathy were more likely to be referred for CA. Presumably some of these patients had tachycardia-induced cardiomyopathy and benefited from maintenance of normal sinus rhythm or decreased burden of AF, as was shown in the CASTLE-AF Trial [24].

We found significant differences in practice patterns unrelated to patient demographics. Patients located within the Northeast and North Central regions were significantly less likely to be referred for early CA. This mirrors geographic variation seen in other studies of CA as well as cardiac devices more generally [20, 26, 27]. A study of Medicare patients found that ablation was more likely in the South and West, although there were more specific differences within referral regions [20]. Even within Europe, studies have shown geographic variation in CA utilization [28]. Further investigation is required to understand the drivers of these differences, which may include physician training networks affecting referral threshold, hospital incentives and payment methods, or patient preferences. Insurance type is also a significant predictor of early ablation. Patients with PPO or high-deductible health plans are more likely to undergo early ablation. Potential explanations include decreased barrier to early electrophysiology referral, different out-of-pocket expense, or fewer barriers for trialing alternative treatments prior to proceeding to ablation.

Our study includes a large, nationally-representative sample of commercially insured patients. Our study focuses on a non-elderly adult cohort, which coincides with our understanding of who is likely to benefit from ablation. Given non-uniform adoption of CA, there are opportunities to explore the causes of these differences to ensure more uniform adoption of early ablation.

### Study limitations

Our study has several limitations. Despite its large size, there is year-to-year variation in total patient encounters due to changes in agreements with the commercial vendor. This limits the ability to characterize absolute procedural volume over time, but still permits understanding of likelihood of undergoing CA. Furthermore, there were differences in geographic representation within the dataset due to vendor agreements, with patients in the South and North Central regions more frequently represented. We cannot draw conclusions about the overall prevalence of ablation within these regions, but the factors associated with early ablation should not be affected. Additionally, we were unable to determine whether ablation was successful in maintaining normal sinus rhythm given we used a claims-based dataset, although future work will explore surrogates of successful ablation including repeat ablation and hospitalizations.

Our reliance on diagnosis codes for AF phenotyping and the underlying criteria for incident AF identification could have influenced the study results. While the type of AF (paroxysmal, persistent, permanent) may relate to the benefit of early ablation, this distinction was only implemented in ICD-10 and does not appear reliable or generalizable given the majority of patients were coded as having unspecified AF. Nonetheless, we would expect this distinction to be less relevant within the first year of diagnosis. As our study includes a younger population, the findings cannot be extended to an older population.

## Conclusions

In conclusion, early referral for CA of AF in a young population increased from 5.0% in 2010 to 10.5% in 2016, with most patients still trialed on an AAD. There is significant geographic variation in utilization of CA, with patients in the Northeast and North Central regions less likely to be referred. Further studies are required to understand the drivers of these differences as well as the impact of early CA on patient outcomes, hospitalization, and treatment cost.

## Data Availability

The data that support the findings of this study are available from IBM Marketscan® database but restrictions apply to the availability of these data, which were used under license and in collaboration with the Healthcare Economics group at Johnson and Johnson for the current study, and so are not publicly available. Data are however available from the authors upon reasonable request and with permission of Johnson and Johnson.

## Abbreviations

AAD: Anti-arrhythmic drug
AF: Atrial fibrillation
CA: Catheter ablation
CCI: Charlson comorbidity index
CDHP-HDHP: Consumer-driven health plan – high deductible health plan
DCCV: Direct current cardioversion
EPO-HPO: Exclusive provider network – health maintenance organization
PPO: Preferred provider organization
